# Degradation of Potassium Rock by Earthworms and Responses of Bacterial Communities in Its Gut and Surrounding Substrates after Being Fed with Mineral

**DOI:** 10.1371/journal.pone.0028803

**Published:** 2011-12-13

**Authors:** Dianfeng Liu, Bin Lian, Bin Wang, Guofang Jiang

**Affiliations:** 1 Jiangsu Key Laboratory for Microbes and Functional Genomics, Jiangsu Engineering and Technology Research Center for Microbiology, College of Life Sciences, Nanjing Normal University, Nanjing, People's Republic of China; 2 State Key Laboratory of Environmental Geochemistry, Institute of Geochemistry, Chinese Academy of Sciences, Guiyang, People's Republic of China; 3 Department of Bio-Engineering, Henan Institute of Science and Technology, Xinxiang, People's Republic of China; University of Wisconsin-Milwaukee, United States of America

## Abstract

**Background:**

Earthworms are an ecosystem's engineers, contributing to a wide range of nutrient cycling and geochemical processes in the ecosystem. Their activities can increase rates of silicate mineral weathering. Their intestinal microbes usually are thought to be one of the key drivers of mineral degradation mediated by earthworms,but the diversities of the intestinal microorganisms which were relevant with mineral weathering are unclear.

**Methodology/Principal Findings:**

In this report, we show earthworms' effect on silicate mineral weathering and the responses of bacterial communities in their gut and surrounding substrates after being fed with potassium-bearing rock powder (PBRP). Determination of water-soluble and HNO_3_-extractable elements indicated some elements such as Al, Fe and Ca were significantly released from mineral upon the digestion of earthworms. The microbial communities in earthworms' gut and the surrounding substrates were investigated by amplified ribosomal DNA restriction analysis (ARDRA) and the results showed a higher bacterial diversity in the guts of the earthworms fed with PBRP and the PBRP after being fed to earthworms. UPGMA dendrogram with unweighted UniFrac analysis, considering only taxa that are present, revealed that earthworms' gut and their surrounding substrate shared similar microbiota. UPGMA dendrogram with weighted UniFrac, considering the relative abundance of microbial lineages, showed the two samples from surrounding substrate and the two samples from earthworms' gut had similarity in microbial community, respectively.

**Conclusions/Significance:**

Our results indicated earthworms can accelerate degradation of silicate mineral. Earthworms play an important role in ecosystem processe since they not only have some positive effects on soil structure, but also promote nutrient cycling of ecosystem by enhancing the weathering of minerals.

## Introduction

Bioweathering as a common geochemical process is the erosion, decay and decomposition of rocks and minerals mediated by living organisms [1], [2]. The bioweathering process plays a fundamental role in the release of nutrients from rocks, and is associated with global climate and environmental changes [1], [3]. Microorganisms, higher plants and animals can weather rock aggregates through biomechanical and biochemical attack on mineral constituents [4]. Among the animals which can degrade rock, earthworms are now known to increase rates of silicate mineral weathering [5], [6], [7], [8], [9].

Earthworms constitute the dominant soil macrofauna in many soils [10], and they had been described as ecosystem engineers due to their key role in altering the biological activity and physical structure of soils through their burrowing and casting and, therefore, contributing to a wide range of nutrient cycling and geochemical processes in soils [11], [12]. Earthworms' role in promoting mineral degradation had been found by many researchers. For example, Suzuki *et al*. examine the breakdown of mineral grains by earthworms, *Eisenia feida*, through feeding experiments, and the results of their study indicated that even 1 day after feeding of mineral grains, the casts of soil animals contained finer, rounded mineral grains that were not included in the initial prepared mineral samples [5]. Carpenter *et al*. investigated the role of earthworms in mineral weathering using the minerals anorthite, biotite, olivine, smectite and kaolinite mixed with a sterilized manure substrate. The changes in mineralogy, analysed using X-ray diffraction, showed that weathering of anorthite, biotite, smectite and kaolinite were accelerated by the earthworms [8]. A few factors may contribute to mineral weathering mediated by earthworms, such as the low pH and bacteria-rich microenvironment in the guts of earthworms, the powerful mechanical grinding action of guts caused by the peristaltic movements used to move food along the gut and the ligands originated from eathworms and their gut microbes [6], [8]. Of these factors, earthworms' gut microbes may play important role in increasing rates of mineral weathering [8]. The earthworms' gut are enriched in microbes, with concentrations much higher than in the surrounding environment. There is therefore greater potential for microbially mediated weathering to occur in the earthworms' gut [8] Alternatively, the increase in microbial activity as a result of earthworms' activity could result in increased weathering rates in surrounding minerals [8].

Potassium (K) is the third major macronutrient for plant growth that may significantly affect the growth and production of crops along with Nitrogen (N) and Phophorus (P) [13], [14], [15]. Many countries, such as China, India and Brazil, are important agricultural countries but deficient in potassium fertilizer resources [16], [17]. These countries are fortunate to be rich in low-grade potassium-bearing rock (PBR) [18], [19]. However, potassium in the structure of silicate can hardly be used by plants when used as fertilizer [20]. Earthworms' role of releasing potassium from silicate minerals has also been found. For instance, Basker *et al.* reported that exchangeable potassium content increased significantly in soil populated by earthworms when compared with the same kind soil devoid of earthworms [21]. They concluded that the increase was due to the release of K from the non-exchangeable K pool as soil material passed through the worm gut.

It is speculated that the microbiota in earthworms' gut may play important role in accelerating the mineral degradation mediated by earthworms [5], [8], [9], [11], [12], [22]. These microbes enhance the weathering of minerals by lowering pH or by producing ion-complexing organic ligands, or by both [23], [24]. The weathering of minerals not only releases essential plant nutrients, such as K, Ca and P, from the mineral structure, but also can increase the cation exchange capacity and water holding capacity of soils through the formation of clay minerals and oxyhydroxides [8], [9]. However, an unanswered question is whether the composition of earthworms' intestinal microbes and their dominant members will change after being fed with mineral powders. Intestinal microbes of earthworms exist in the form of “communities”, and these communities contribute the weathering of natural minerals by developing a microbial ecosystems metabolic network [25]. Characterizing the gut microbial communities of earthworms will help us to understand the mechanisms of earthworms' weathering PBR and get a better understanding of those microbial communities which may be involved in the degradation of PBRP.

## Materials and Methods

### Ethics Statement

The earthworms and potassium-bearing rock for this study were collected in public land in Qixia district, Nanjing region, China, and their collections and the study were approved by the Chinese government.

### Sample description

The earthworms *Pheretima carnosa* and soil were collected manually in April of 2009in the campus of Nanjing Normal University (Qixia district, Nanjing region, China). This type of soil is called “ yellow-brown soil ”.

PBR was collected from Fuquan region, Guizhou Province, China. The mineral constitution and the element composition of the rock were determined separately using X-ray diffraction (XRD) (Rigaku, D/MAx-2200) and X Ray Fluorescence (XRF) (Axios, PW4400) in the Institute of Geochemistry, Chinese Academy of Sciences. The rock's mineral composition contains feldspar (75.52%), mica(9.25%), dolomite(7.15%), Montmorillonite(4.10%), Kaolinite(3.46%) and Hornblende(0.52%). The major element oxides of the PBR is K_2_O(9.67%), Al_2_O_3_(18.06%), SiO_2_(57.75%), Fe_2_O_3_(5.3%), CaO(0.21%), MgO(3.21%), Na_2_O(0.14%) and LOI(4.98%). The rock was crushed and sieved to obtain powders of 250 to 500 µm for feeding experiments.

### Feeding experiment

The feeding experiments were carried out in the plastic container of 35 cm in diameter and 40 cm in depth. Earthworms were fed only with soil for ten days to remove any preexisting organic matters from their digestive system (Treatment 1). Subsequently, three treatments were performed:

Treatment 2—Earthworms and mixture of PBRP and soil, at the ratio of 4∶6.
Treatment 3—Earthworms and PBRP.
Treatment 4—PBRP only, no earthworm.

About 500 g substrate (soil, PBRP or soil plus PBRP) were placed in every container. The ratio for PBRP: soil in treatment 2 is based on the composition of the compost enriched by PBRP we developed previously. The earthworm treatments comprised six earthworms per container. Sterile deionized water was added to the substrate to maintain appropriate moisture for the growth of earthworms. During feeding earthworms, the substrate moisture content was at approximately 25%. The plastic containers were covered to avoid light and the laboratory temperature was about 25°C throughout the experiment. In pre-experiment, some earthworms were found to become dead after feeding with PBRP for two weeks. Dead earthworm would affect the compositions of bacterial communities of the PBRP and soil processed by the animal. Therefore, all treatments were sampled after ten days. The substrates in each box were homogenized before sampling. Earthworms were sedated, surface sterilized with ethanol (75%) and dissected under sterile conditions. The whole gut content of each specimen without gut wall was extracted with a sterile spatula and transferred into sterile 1.5 mL tubes [26]. Each gut sample was used in electron microscopy studies and DNA extraction.

### Examination of samples by transmission electron microscope

Seven samples were air-dried and examined using transmission electron microscope (TEM) to observe the morphology of the earthworms' gut content, the PBRP and the soil sample. TEM measurements were carried out using a Hitachi H-7650 TEM system (Japan) at a working voltage of 80 kV.

### Determination of water-soluble and HNO_3_-extractable K Al, Fe and Ca

To examine the influence of earthworms on mineral degradation, concentrations of water-soluble and HNO_3_-extractable K, Al, Fe and Ca of three samples, including the original prepared PBRP (number: 0K), the PBRP after being fed to earthworms for ten days (number: 10EK, from treatment 3) and the control PBRP which was incubated at proper humidity level (about 25%) for ten days without earthworms (number: 10K, from treatment 4), were determined by inductively coupled plasma atomic emission spectrometry (ICP-AES, Optima 2100 DV, Perkin Elmer, USA).

Determination of concentration of water-soluble K, Al, Fe and Ca was performed using the methods as previously described [19], [27], [28] with the following modifications: Five gram of dried sample was mixed with 50 ml of ddH_2_O in a 250 ml flask followed by 30 minutes of vigorous shaking. After shaking, the samples were centrifuged at 8000 rpm for 10 minutes. The supernatants were filtered through 0.45 µm filter paper to obtain a purified extract. The concentration of water-soluble K, Al, Fe and Ca in the purified extract were determined by ICP-AES.

Concentration of nitric acid-extractable K Al, Fe and Ca were determined using the previously described methods [19], [27], [28] with the following modifications: Five gram of dried sample was added to a 150 ml triangular flask containing 50 ml of 1 N HNO_3_, and boiled for 10 minutes. The digested mixture was transferred to a 250 ml volumetric flask and diluted with water to 250 ml. Subsequently, the extract was purified by centrifuging and filtering to determine its concentration of HNO_3_-extractable K, Al, Fe and Ca by ICP-AES.

### DNA extraction

The samples for molecular analysis of microbial populations consisted of the soil after being fed to earthworms for ten days (number:10ES, from treatment 1), the gut content of the earthworms fed with soil for ten days (number:10GS, from treatment 1) the original prepared PBRP (number: 0K), the PBRP after being fed to earthworms for ten days (number: 10EK, from treatment 3) and the gut content of the earthworms fed with PBRP for ten days (number: 10GK, from treatment 3). DNA was extracted using a Soil Extraction Kit (OMega, US) according to the manufacturer's instructions. The crude DNA was purified through a minicolumn purification method [29] and quantified by ethidium bromide-UV detection on an agarose gel.

### PCR Amplification of Bacterial 16S rRNA Genes

The 16S rRNA gene fragments were amplified by PCR using a Whatman Biometra thermo-cycler (Göttingen, Germany). Each PCR mixture contained 0.4 mM of deoxynucleoside triphosphates, 0.4 µM of each primer, 3 µl of 10× PCR buffer, 2 mM magnesium chloride, 1 U of Taq DNA polymerase and 1 µl (about 5–15 ng) of template DNA in a final volume of 30 µl. Amplification was made using a touchdown protocol. The Primers used for amplifications were 16S-fD1 (5′-AGAGTTTGATCCTGGCTCAG-3′) and 16S-rD1 (5′-ACGGTTACCTTGTTACGACTT-3′) [30]. PCR was performed with the following procedure: the annealing temperature was set at 65°C and was decreased by 1°C for every cycle until reaching a “touchdown” at 55°C. The amplification program consisted of 5 min at 94°C, and 10 touchdown cycles of denaturation at 94°C for 1 min, annealing at 65°C (with the temperature decreasing 1°C each cycle) for 1 min, and extension at 72°C for 2 min, followed by 25 cycles of 94°C for 1 min, 55°C for 1 min, and 72°C for 2 min. During the last cycle, the length of the extension step was increased to 10 min. After amplification, PCR products were analyzed by electrophoresis in 1.5% (w/v) agarose gels.

### Clone library construction and restriction digestion of 16S rRNA gene

PCR products were excised from 2% low melting agarose (Sigma, St-Louis, MO) and the DNA was purified using a Gel Isolation Kit following manufacturer's instructions (Promega, Madison, WI, USA). Purified amplicons were then ligated into a pGEM-T Easy vector and transformed into competent *E. coli* DH5α cells according to the manufacturer's instructions (Promega, Madison, WI, USA). The transformed cells were selected on LB agar medium containing Ampicillin, X-gal, and IPTG, and incubated at 37°C overnight. The recombinant white colored colonies were screened for inserts by colony PCR using the primers T7 (5′-TAATACGACTCACTATAGGG-3′) and SP6 (5′-ACGATTTAGGTGACACTATAG-3′) [31]. The products of colony PCR were digested in 10 µl reaction volumes with 1 U of *Hinf* I and 1×buffer (Takara, Japan) for 4 h at 37°C. The resulting RFLP (restriction fragment length polymorphism) fragments were separated by gel electrophoresis in 2.0% agarose. Bands were visualized by staining with ethidium bromide and UV illumination. Clones were grouped into operational taxonomic units (OTU) based on the RFLP patterns. One representative clone from each group was chosen for partial 16S rRNA gene sequencing. Sequencing was performed by Shanghai Sangon Biological Engineering Technology & Services Co., Ltd.

### Statistical analyses for bacterial communities

Statistical analyses for bacterial communities were done as described previously [32], [33]. Each OTU was considered a separate species for statistical analyses. Shannon-Weiner index (*H′*) and Simpson index (*D*) were used to summarize the diversity of a bacterial community, represented respectively by 
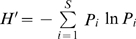
 and 
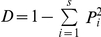
, where *S* is the observed number of taxa and *P*_i_ is the frequency of the *i*th species. Evenness index (*E*) derived from Shannon-Weiner index was calculated with the equation 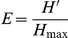, where *H*_max_ = ln*S* which occurs when all species are present in equal number. Coverage (*C*) was calculated with the equation 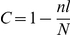, where *nl* is the number of unique OUT observed and *N* is the total number of OTUs (i.e., the sum of unique OTUs plus OTUs observed more than once). This coverage value assessed the amount covered from the sampled population and may not necessarily reflect the full diversity of the organisms [33].

### Phylogenetic analysis

The obtained 16S rRNA gene sequences were compared to the National Center for Biotechnology Information database using BLAST to find very similar sequences in NCBI's databases [34]. Those similar sequences were downloaded from Genbank, and aligned using ClustalW2 [35] with parameters set to default. Chimeric sequences were removed based on the BLAST, ClustalW2 and Chimera Check program results [36]. The data sets without the chimeric sequences were aligned using ClustalW2 again, the regions of uncertain alignment were omitted, and then the data sets were used to construct molecular phylogenetic trees. Neighbor joining (NJ) criteria was used to determine the relationships among sequences. For the NJ analysis, the Tamura-Nei's nucleotide substitution model was selected with pairwise deletion of gaps. Meanwhile, bootstrap reassembling analysis based on 1000 replicates was used to assess the confidence values attached to the individual nodes.

### Microbial community clustering with UniFrac

A phylogenetic tree, containing only the 16S rRNA sequences from this study, using an archaea as outgroup was exported from PAUP 4.0b10 [37], and an environment file, linking each sequence to the environment that it came from, was created manually. Information in the phylogenetic tree and the environment file was used to measure the difference between bacterial communities in samples by using the UniFrac method [38], [39]. We use an hierarchical clustering method, called unweighted pair group method with arithmetic averages (UPGMA), to cluster the community samples with unweighted and weighted UniFrac. The confidence in the nodes of the UPGMA tree was assessed by 100 jackknife resamplings. Principal coordinates analysis (PCA) was performed by using unweighted and normalized weighted UniFrac test.

## Results

### Effect of earthworms on the degradation and morphology of PBRP

**Figure 1:**
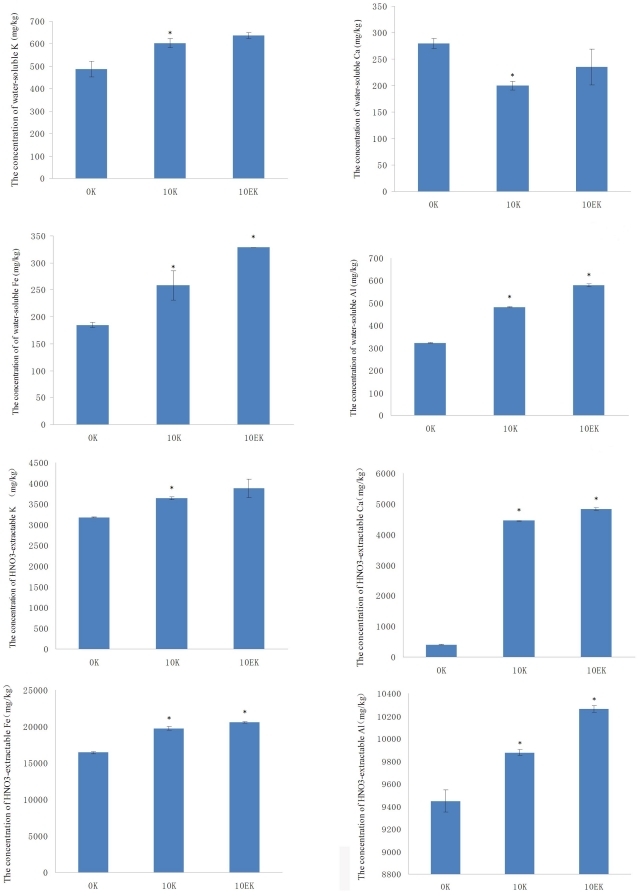
The amounts of water-soluble and HNO_3_-extractable K Al, Fe and Ca released from PBRP. The numbers 0K, 10K and 10EK mean the original prepared PBRP, the PBRP incubated at proper humidity level for ten days without earthworms and the PBRP after being fed to earthworms for ten days, respectively. Error bars are ± standard deviation (n = 2). The asterisks (*) above 10K and 10EK denote the value significantly greater than the values of 0K and 10K (p<0.05), respectively.

The effect of earthworms on the degradation of PBRP was observed through changes in the concentration of water-soluble and HNO_3_-soluble elements (Figure 1). The results of ANOVA and post hoc tests for analysing for the concentrations of water-soluble and nitric acid-extractable K, Al, Fe and Ca of the samples, including 0K, 10K and 10EK, were showed by table 1 and 2. The PBRP had some increases in water-soluble K, Fe and Al after it was incubated at proper humidity level for ten days. After earthworms activity, there were more increases in the three water-soluble elements. However, decrease in the water-soluble Ca from 10K and 10EK was observed compared to the 0K. Statistical analysis indicated that the concentrations of water-soluble Fe and Al were significantly (p<0.05) increased after 10d from the inoculation with earthworms, comparing to the control without earthworms (10K). In addition, the concentrations of HNO_3_-extractable K, Ca, Fe and Al of the PBRP, incubated at proper humidity level for ten days, had also some increases, and earthworms' activity cause the PBRP to have more increase in the four HNO_3_-extractable elements. Statistical analysis showed that there were significant increase (p<0.05) in the concentrations of HNO_3_-extractable Ca, Fe and Al in the PBRP incubated with earthworms, compared with the unincubated control (10K). The results indicated that the activity of earthworms could indeed accelerate the degradation of the PBRP compared to the control without earthworms.

**Table 1:** Analysis of variance for the concentrations of water-soluble and nitric acid-extractable K, Al, Fe and Ca of the samples.

Elements		Sum of Squares	df	Mean Square	F	Sig.
Water-soluble	K	24659.560	2	12329.780	20.697	0.018
	Ca	6334.943	2	3167.472	7.379	0.069
	Fe	20810.230	2	10405.115	41.853	0.006
	Al	67093.043	2	33546.522	2002.777	0.000
HNO_3_-extractable	K	514530.333	2	257265.167	15.368	0.027
	Ca	24110569.000	2	12055284.500	19671.392	0.000
	Fe	19382797.000	2	9691398.500	372.474	0.000
	Al	664100.333	2	332050.167	90.473	0.002

**Table 2:** Post hoc tests for analyzing the concentrations of water-soluble and nitric acid-extractable K, Al, Fe and Ca of the samples.

Elements	(I) Sample	(J) Sample	Mean Difference (I–J)	Std. Error	Sig.
Water-soluble K	10K	0K	115.7	24.40748	0.018
	10EK	0K	149.8	24.40748	0.009
		10K	34.1	24.40748	0.257
Water-soluble Ca	10K	0K	−79.35	20.71895	0.031
	10EK	0K	−45.05	20.71895	0.118
		10K	34.3	20.71895	0.196
Water-soluble Fe	10K	0K	73.4	15.76737	0.019
	10EK	0K	144.25	15.76737	0.003
		10K	70.85	15.76737	0.021
Water-soluble Al	10K	0K	159.2	4.09268	0.000
	10EK	0K	256.55	4.09268	0.000
		10K	97.35	4.09268	0.000
HNO_3_-extractable K	10K	0K	465	129.38444	0.037
	10EK	0K	705.5	129.38444	0.012
		10K	240.5	129.38444	0.160
HNO_3_-extractable Ca	10K	0K	4050.5	24.75547	0.000
	10EK	0K	4429	24.75547	0.000
		10K	378.5	24.75547	0.001
HNO_3_-extractable Fe	10K	0K	3313	161.30406	0.000
	10EK	0K	4167.5	161.30406	0.000
		10K	854.5	161.30406	0.013
HNO_3_-extractable Al	10K	0K	430	60.58190	0.006
	10EK	0K	814.5	60.58190	0.001
		10K	384.5	60.58190	0.008

**Figure 2:**
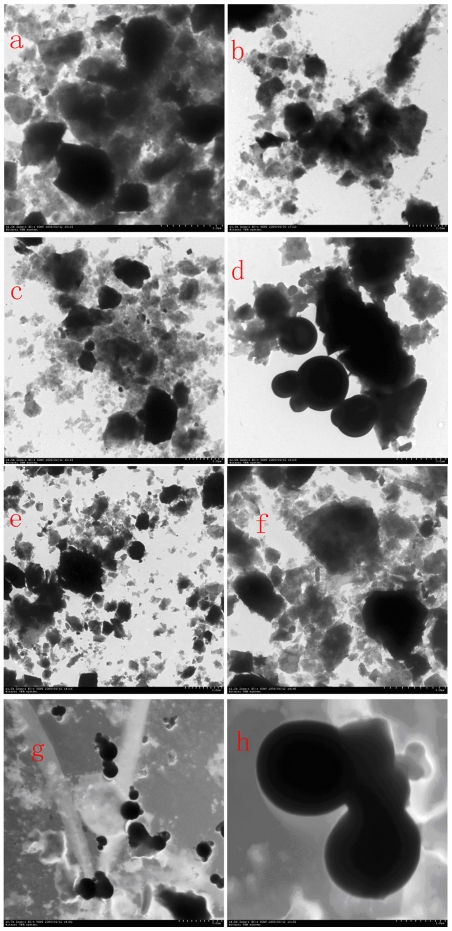
TEM images are of the soil, the PBRP and the gut's contents of earthworms feeding on PBRP and soil. Among these images, a and b showed the TEM images of the soil and the gut's content after feeding earthworms with soil for ten days, respectively; c and d showed that of the mixture of PBRP and soil and the gut's content after being fed with the mixture respectively; e showed the images of fresh PBRP; f is the images of the surrounding PBRP after being fed; g and h showed the images of the gut's content after being fed with PBRP, respectively.

The TEM images of earthworms' gut contents and surrounding substrates, including PBRP and soil, were showed by Figure 2. The observation of various soil samples under TEM indicated that the morphology of soil minerals from earthworms' gut was similar to the surrounding soil (Figure 2-a and 2-b), and the initially prepared PBRP and those after being fed to earthworms for ten days had irregular shape and dense structure (Figure 2-e and 2-f). However, many round pellets, along with the soil grains, were observed in the gut content of the earthworms fed with the mixture of PBRP and soil (Figure 2-d). Besides, no irregular particles were observed in the gut content of the earthworms fed with the PBRP, all the pellets were round in the gut of earthworms fed with the minerals (Figure 2-g and 2-h). Whenas, no round pellets were observed in the gut of the earthworms fed with soil alone, the surrounding mineral grains and the surrounding soil. Therefore, the round pellets could be the PBRP ingested by the earthworms.

### RFLP analysis of 16S rRNA clone libraries

Five clone libraries of bacterial PCR products were constructed for 10ES, 10GS, 0K, 10EK, 10GK. The 10ES library contained 391 clones and its coverage value was 96.42%. The clones of 10ES library were grouped into 33 OTUs based on their RFLP profiles. The 10GS library consisted of 383 clones and its coverage value was 97.91%. The clones of 10GS library were classified into 31 OTUs according to the RFLP profiles. The 0K library contained 10 OTUs (out of 261 clones) and its coverage value was 98.08%. The 10EK library was comprised of 289 clones, including 29 OTUs, and its coverage value was 99.31%. The 10GK library consisted of 335 clones, contained 34 OTUs and its coverage value was 97.91%.

Bacterial Diversity indexes from RFLP analysis are showed in table 3. The PBRP (0K) had severely low bacterial diversity index; however, other samples had much higher values than it. For instance, Shannon-Wiener indexes of 10ES, 10GS, 10EK and 10GK were 1.8 to 2.3 times more than that of 0K. The bacterial diversities in the guts of the earthworms fed with PBRP and the PBRP after earthworms' action were even higher than the ones in the guts of the earthworms fed with soil and the soil after earthworms' action, showing that many microorganisms can survive on the surface of PBRP in the presence of earthworms and optimal amount of water in the mineral grains.

**Table 3:** Bacterial diversity indexes from the five samples.

Sample number	Shannon-Wiener *H′*	Simpson *D*	Evenness *E*	Coverage *C*
10ES	2.47	0.873	0.71	96.42%
10GS	2.49	0.865	0.72	97.91%
0K	0.87	0.44	0.38	98.08%
10EK	2.91	0.931	0.86	99.31%
10GK	2.78	0.916	0.69	97.91%

### Bacterial community structure

**Figure 3:**
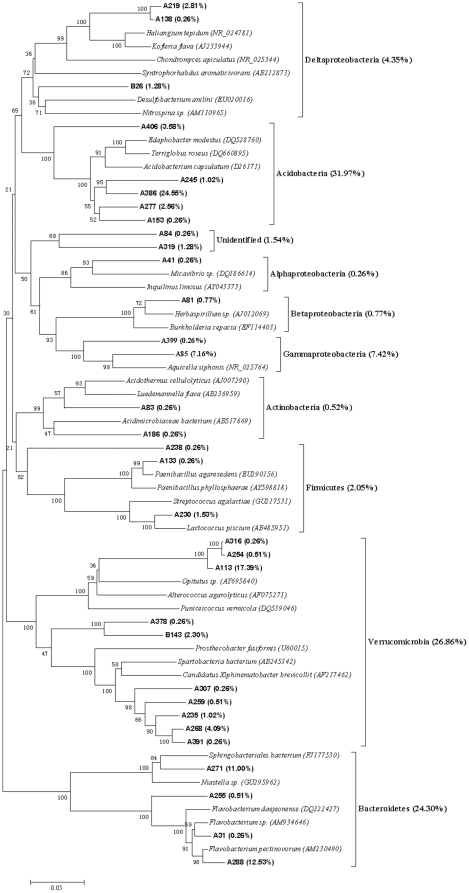
Neighbour-joining tree of 16SRNA gene sequences depicting the phylogenetic relationships of clones from the soil samples after having been processed by the earthworms in ten days. The scale bars represent a 5% sequence divergence and percentages of 1000 bootstrap resamplings are shown at the nodes. Species names and OTUs' numbers are followed by their GenBank accession numbers their proportion in all clones of the library, respectively.

**Figure 4:**
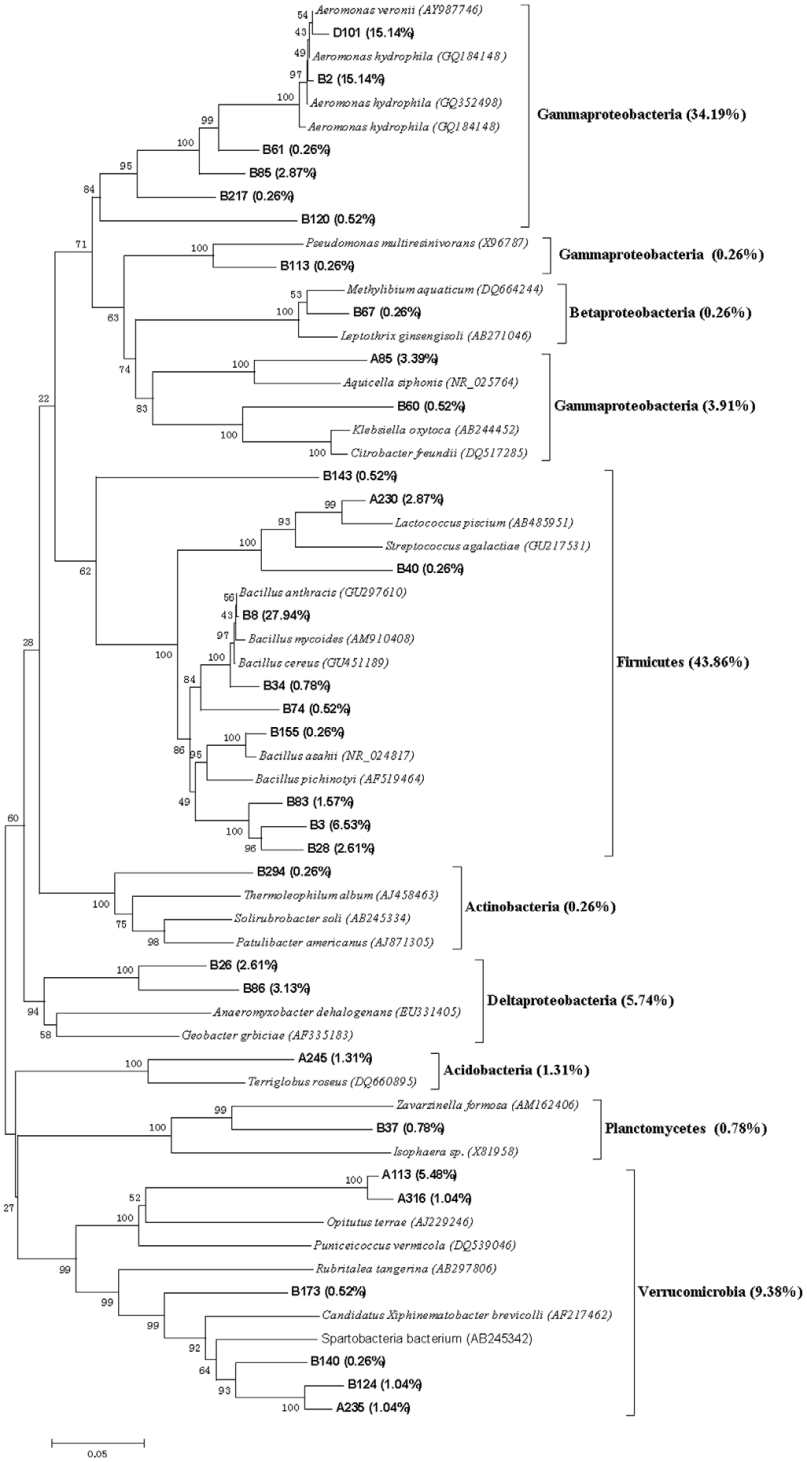
Neighbour-joining tree of 16SRNA gene sequences depicting the phylogenetic relationships of clones from the gut contents of the earthworms fed with soil for ten days. The scale bars represent a 5% sequence divergence and percentages of 1000 bootstrap resamplings are shown at the nodes. Species names and OTUs' numbers are followed by their GenBank accession numbers their proportion in all clones of the library, respectively.

**Figure 5:**
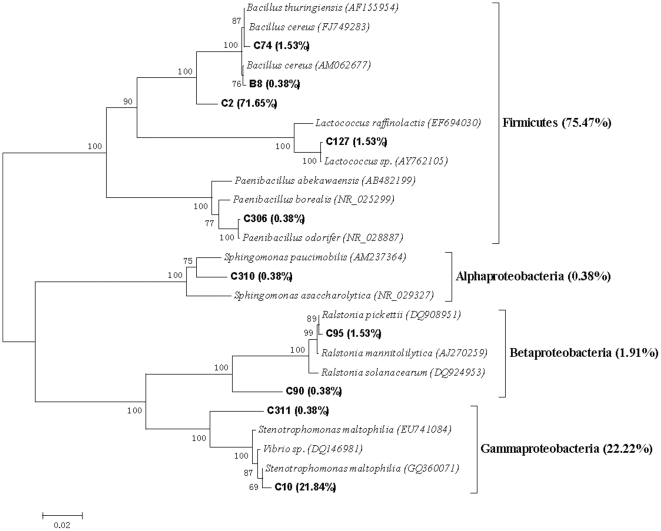
Neighbour-joining tree of 16SRNA gene sequences depicting the phylogenetic relationships of clones from the natural PBRP sample. The scale bars represent a 2% sequence divergence and percentages of 1000 bootstrap resamplings are shown at the nodes. Species names and OTUs' numbers are followed by their GenBank accession numbers their proportion in all clones of the library, respectively.

**Figure 6:**
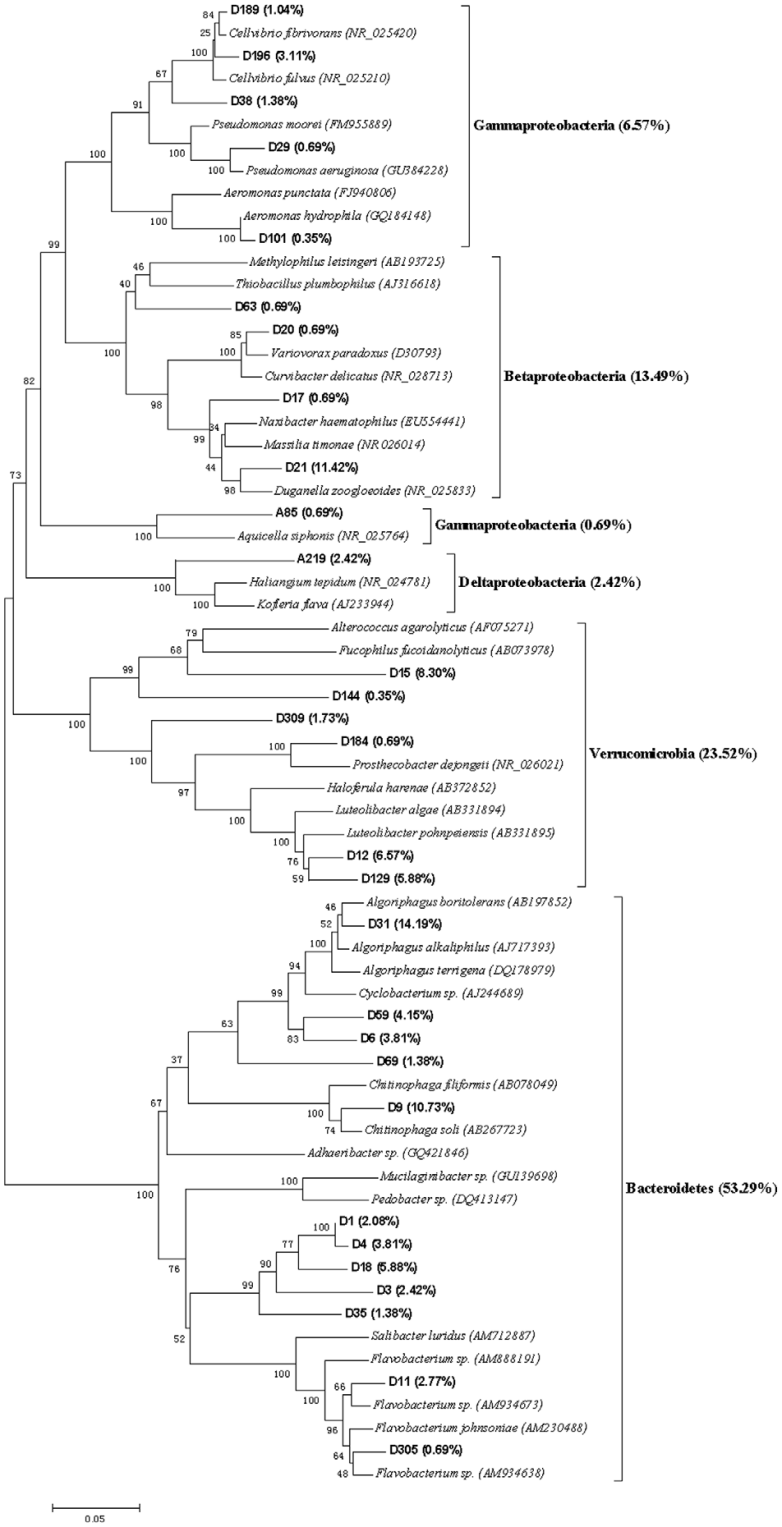
Neighbour-joining tree of 16SRNA gene sequences depicting the phylogenetic relationships of clones from the PBRP samples after having been processed by the earthworms in ten days. The scale bars represent a 5% sequence divergence and percentages of 1000 bootstrap resamplings are shown at the nodes. Species names and OTUs' numbers are followed by their GenBank accession numbers their proportion in all clones of the library, respectively.

**Figure 7:**
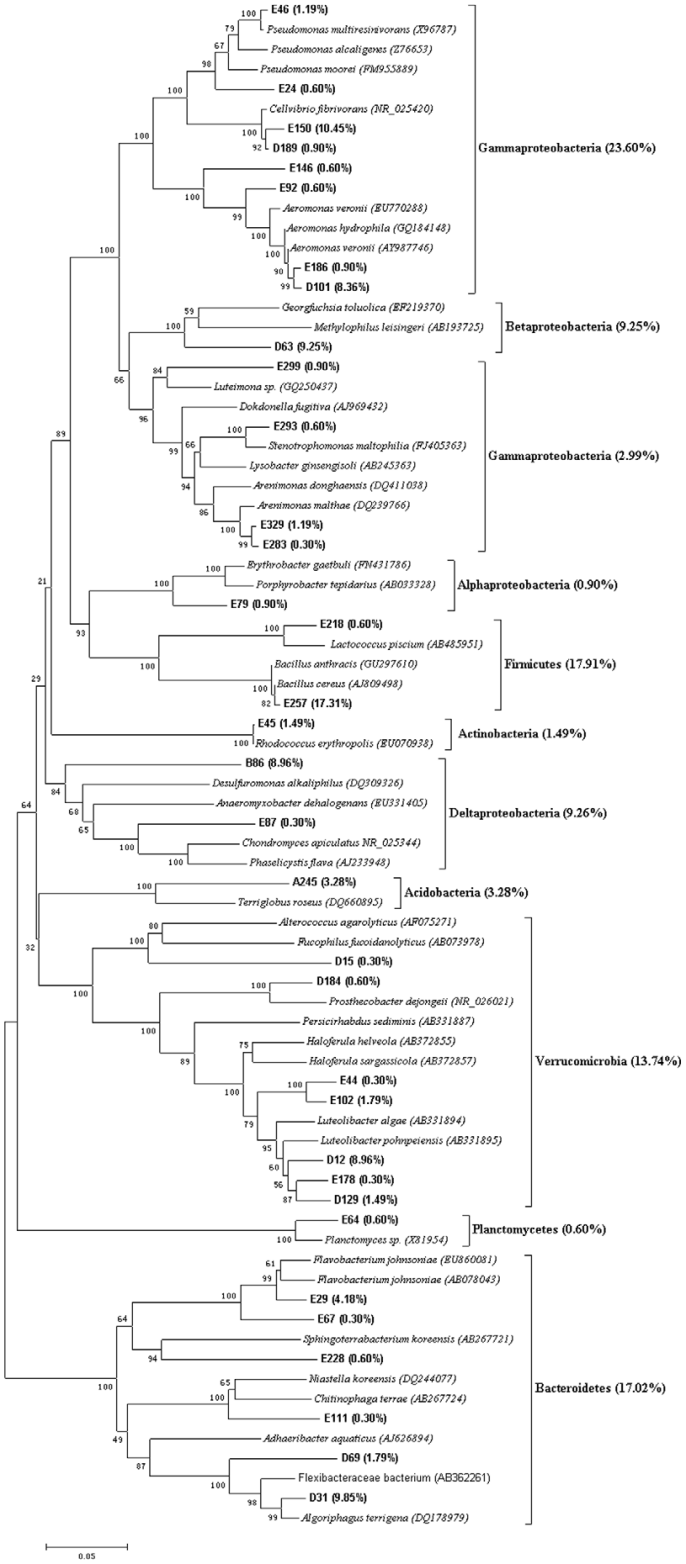
Neighbour-joining tree of 16SRNA gene sequences depicting the phylogenetic relationships of clones from the gut contents of the earthworms fed with PBRP for ten days. The scale bars represent a 5% sequence divergence and percentages of 1000 bootstrap resamplings are shown at the nodes. Species names and OTUs' numbers are followed by their GenBank accession numbers their proportion in all clones of the library, respectively.

The representative clones of each clone library were sequenced based on the RFLP profiles. Eleven chimeric sequences were found and discarded in the subsequent analysis. The Clone sequences were deposited with the accession numbers HM459607 to HM459720. More than 95% similarity was found between the sequences after BLAST search in the Genbank. The results from the data sets of representative sequences from each library showed the C+G content was higher compared to the A+T in all data sets. Therefore, the Tamura-Nei model was selected to construct molecular phylogenetic trees, which not only considered transversions and transitions but also the frequency of A, T, C and G. The phylogenetic trees, obtained using the NJ method based on the 16S rRNA sequences of each clones are showed in figure 3,4,5,6,7, respectively.

Figure 3 is the phylogenetic tree based on the 16S rRNA gene sequences of the clones obtained from the soil in which earthworms fed with ten days. It revealed that the library contained sequences homologous to *Verrucomicrobia* (26.86%), *Bacteroidetes* (24.30%), *proteobacteria* (12.8%) including *Alphaproteobacteria* (0.26%), *Betaproteobacteria* (0.77%), *Gammaproteobacteria* (7.42%) and *Deltaproteobacteria* (4.35%), *Firmicutes* (2.05%), *Actinobacteria* (0.52%) and two unidentified OTUs. In the OTUs of the library, the first dominant OTU A386 constituted 24.55% of the library clones, the second dominant OTU A113 constituted 17.39% of the library clones, the third dominant OTU A288 constituted 12.53% of the library clones, and the other two dominant OTUs, A271and A85, constituted 11.00% and 7.16%, respectively. The results of the phylogenetic tree and homologous sequences indicated that A386 belonged to *Acidobacteriaceae* (*Acidobacteria; Acidobacteriales*), A113 was a member of *Opitutae* group (*Verrucomicrobia*), A288 was *Flavobacterium* sp. (*Bacteroidetes*; *Flavobacteria*; *Flavobacteriales*; *Flavobacteriaceae*), A271 belonged to *Sphingobacteriales* (*Bacteroidetes*, *Sphingobacteria*), and A85 was *Aquicella* sp. (*Proteobacteria*; *Gammaproteobacteria*; *Legionellales*; *Coxiellaceae*).

Figure 4 shows the phylogenetic tree of 16S rRNA sequences from the intestinal representative bacteria of earthworms fed with soil. The sequences of the library had similarities to *proteobacteria* (44.36%) including *Gammaproteobacteria* (38.36%), *Betaproteobacteria* (0.26%) and *Deltaproteobacteria* (5.74%), *Firmicutes* (43.86%), *Verrucomicrobia* (9.38%), *Acidobacteria* (1.31%), *Planctomycetes* (0.78%) and *Actinobacteria* (0.26%). The dominant sequence types of this library were positioned within the *Gammaproteobacteria* group and *Firmicutes* group, which was very different from the dominant sequence types of the library of the soil with which earthworms were fed. The five dominant OTUs of the gut content library were B8, B2, D101, B3 and A113, and they constituted 27.94%, 15.14%, 15.14%, 6.53% and 5.48% of the clones of the gut content library, respectively. The results of phylogeny and homologous sequences showed that both B8 and B3 belonged to *Bacillus* (*Firmicutes*; *Bacillales*; *Bacillaceae*) and B8 should be *Bacillus cereus*, and B2 and D101 were associated with *Aeromonas* (*Proteobacteria*; *Gammaproteobacteria*; *Aeromonadale*; *Aeromonadaceae*), and A113 was attributed to *Opitutae* (*Verrucomicrobia*).

The phylogenetic tree created using the 16S rRNA sequences from the original prepared PBRP is given in Figure 5. The phylogenetic analysis showed that the clones in the library belonged to *Firmicutes* (75.09%) and *Proteobacteria* (24.01%). Among the phylum of *Proteobacteria*, *Gammaproteobacteria*, *Betaproteobacteria* and *Alphaproteobacteria* constituted 22.22%, 1.91% and 0.38%, respectively. Many *Firmicutes* are known to produce endospores that are resistant to desiccation and other extreme conditions. In the OTUs of the library, the first dominant OTU C2 constituted 75.09% of the library clones and belonged to *Bacillus* (*Firmicutes*) group based on the phylogenetic analysis. The second dominant OTUs C10 constituted 21.84% of the library clones and belonged to *Stenotrophomonas* (*Proteobacteria*; *Gammaproteobacteria*; *Xanthomonadales*; *Xanthomonadaceae*).

The molecular phylogenetic tree of the representative clone library obtained from PBRP fed to earthworms for ten days is shown in Figure 6. The molecular phylogenetic tree revealed that the library comprised of *Bacteroidetes* (53.29%), *Verrucomicrobia* (23.52%) and *Proteobacteria* (23.12%). Among the phylum of *Proteobacteria*, *Betaproteobacteria*, *Gammaproteobacteria* and *Deltaproteobacteria* constitute 13.49%, 7.26% and 2.42%, respectively. There were four dominant OTUs in the library, D31 (14.19%), D21 (11.42%), D9 (10.73%) and D15 (8.30%). The systematic positions of these dominant OTUs were as following: D31 was a member of *Algoriphagus* group (*Bacteroidetes*; *Sphingobacteriales*; *Sphingobacteria*; *Cyclobacteriaceae*), D21 was closely related to *Duganella* group (*Proteobacteria*; *Betaproteobacteria*; *Burkholderiales*; *Oxalobacteraceae*), D9 was a member of *Chitinophaga* group (*Bacteroidetes*; *Sphingobacteria*; *Sphingobacteriales*; *Chitinophagaceae*), and D15 belonged to *Verrucomicrobia*.

The phylogenetic tree created using 16S rRNA sequences from the intestinal representative bacteria of earthworms fed with PBRP for ten days is shown in Figure 7. The phylogenetic tree showed that the bacterial clone library comprised of seven phyla, including *Proteobacteria* (46%), *Firmicutes* (17.91%), *Bacteroidete* (17.02%), *Verrucomicrobia* (13.74%), *Acidobacteria* (3.28%), *Actinobacteria* (1.49%) and *Planctomycetes* (0.60%). No single OTU dominated the gut content library, but the largest number of clones were from E257 (17.31%), E150 (10.45%), D31 (9.85%), D63 (9.25%), B86 (8.96%), D12 (8.96%) and D101 (8.36%). Among these dominant OTUs, D31, D63, D12 and D101 was also found in the PBRP after being fed to earthworms for ten days (10EK library), and D31, as a dominant OTU of the 10EK library, accounted for 14.19% of the 10EK library and was a member of *Algoriphagus* group. Though D63, D12 and D101 existed in the 10EK library, they were not the dominant OTUs of the library. However, D101 accounted for 15.14% of the 10GS library from the gut content of the earthworms fed with soil for ten days. The results of phylogeny and similarity of sequences showed that D31 belonged to *Algoriphagus* group (*Bacteroidetes*; *Sphingobacteriales*; *Sphingobacteria*; *Cyclobacteriaceae*), D63 belonged to *Betaproteobacteria*, B86 was closely related to *Deltaproteobacteria* (*Proteobacteria*), D12 was a member of *Luteolibacter* group (*Verrucomicrobia*; *Verrucomicrobiae*; *Verrucomicrobiales*; *Verrucomicrobiaceae*), D101 was associated with *Aeromonas sp*. (*Gammaproteobacteria*; *Aeromonadales*; *Aeromonadaceae*), E257 was a member of *Bacillus cereus* group (*Firmicutes*; *Bacillales*; *Bacillaceae*), and E150 belonged to *Cellvibrio* (*Proteobacteria*; *Gammaproteobacteria*; *Pseudomonadales*; *Pseudomonadaceae*).

The number of shared OTUs between the five samples is showed in Table 4. Though the phylogenetic analysis revealed a strong occurrence of *Gammaproteobacteria* in the two gut samples, the statistical analysis of Table 4 revealed that the gut microbiota was influenced by the food source and was considerably different from that of the surrounding substrate. The microbial communities of the five clone libraries were different from each other, but there was evidence of shared OTUs between the library of earthworms gut content and that of surrounding substrates. The number of OTUs, shared by the 10ES library from the soil after being fed to earthworms for ten days and the 10GS library from the gut content of the earthworms fed with soil for ten days, accounted for 24.24% of the 10ES library OTUs and 25.81% of the 10GS library OTUs, respectively. The number of OTUs, shared by the 10EK library from the PBRP after being fed to earthworms for ten days and the 10GK library from the gut content of the earthworms fed with PBRP for ten days, accounted for 31.03% of the 10ES library OTUs and 26.47% of the 10GS library OTUs respectively. Though there were few bacteria in the original prepared PBRP (number: 0K), they had abundant bacterial colonies after feeding earthworms with the mineral grains for ten days and some of the bacteria species were the same as the ones of the gut of the earthworms. The results suggested that most microorganisms of the 10GK library were from the gut of earthworms.

**Table 4:** The number of shared OTUs between the five samples.

number	10ES	10GS	0K	10EK
10GS	8			
0K	0	1		
10EK	2	1	0	
10GK	1	1	0	9

**Figure 8:**
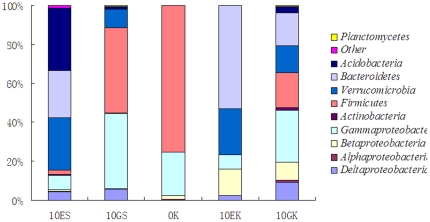
Distribution of clone numbers of each phylogenetic affiliations in each 16S rRNA gene clone library.

Figure 8 showed the difference in clone numbers of each phylogenetic affiliations among the above five 16S rRNA gene clone libraries. The numbers of clones from *Gammaproteobacteria* in the two gut samples were higher than those in the two surrounding substrate samples, respectively. The result indicated that some members from *Gammaproteobacteria* were dominant bacteria in earthworms' gut.

### Comparing of microbial community using unweighted and weighted UniFrac

**Figure 9:**
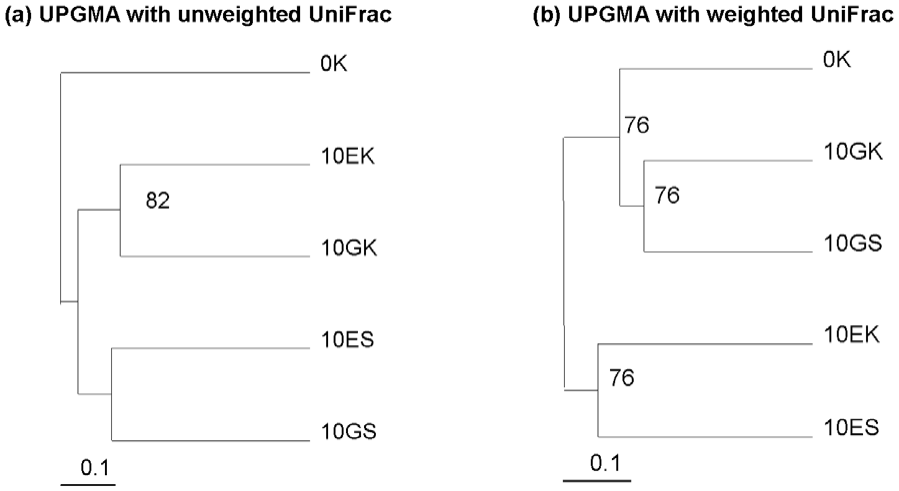
Hierarchical clustering of the five samples with weighted and unweighted UniFrac. The percentage support for nodes supported at least 70% of the time with sequence jackknifing is indicated.

In dendrogram from UPGMA cluster analysis with unweighted UniFrac (Figure 9-a), the two samples from the mineral powder as feeding substrate resemble each other, the two samples from the soil as feeding substrate clustered each other, and the sample 0K, fresh potassium-bearing rock powder, was an independent clade. However, in UPGMA dendrogram with weighted UniFrac (Figure 9-b), the two samples from surrounding substrate clustered into a clade, and the two samples from earthworms gut resembled each other and then grouped into a clade with 0K. This association was well supported by jackknife values.

**Figure 10:**
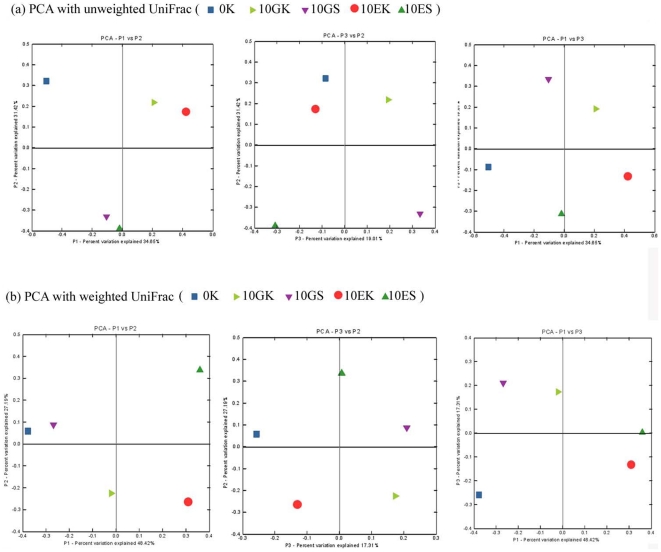
UniFrac unweighted principal coordinate analyses (PCA) (a) and weighted PCA (b) for the five samples.

In principal coordinate analysis with unweighted UniFrac (Figure 10-a), PCA axis 1 showed 34.65% of the variation, PCA axis 2 showed 31.42% of the variation and PCA axis 3 showed 19.01% of the variation. The separation between the soil and meniral powder samples can be most easily seen in PC axes 1 differentiate along 2. It was accordant with the result of UPGMA cluster analysis with unweighted UniFrac. In principal coordinate analysis with weighted UniFrac (Figure 10-b), PCA axis 1 described 48.42% of the variation, PCA axis 2 described 27.19% of the variation and PCA axis 3 described 17.31% of the variation. The gut and surrounding substrate samples differentiated along PC axes 1. The result was consistent with UPGMA cluster analysis with weighted UniFrac.

## Discussion

### Degradation of PBRP mediated by earthworms

Many researchers proposed that the earthworms might mechanically break down mineral grains through ingestion and digestion of soil fractions [5], [7]. For example, Suzuki *et al.* fed earthworms with quartz and feldspar grains, and their results showed that earthworms have an influence on the physical degradation of mineral grains. The ingested grains in the earthworms casts were finer and rounder than the non-ingested grains [5]. Carpenter *et al.* using X-ray diffraction, proved that some minerals are indeed weathered by earthworms [8]. Our results indicated significant increase in the concentration of water-soluble Fe, Al and HNO_3–_extractable Ca, Fe, and Al of PBRP after being fed to earthworms. This proved that earthworms could indeed accelerate PBRP degradation. After PBRP was wetted by sterile deionized water, some microbes could exist on the surface of the mineral. With living of these microbes, the mineral grains were to some extent weathered by them. When the mineral grains were fed to earthworms, there were more microbes on their surface due to earthworms' activities such as burrowing, digesting, casting and excreting. The microorganisms used the nutrients released by weathering mineral to thrive, thus promoting the mineral grains degradation.

The change in water-soluble Ca of 10K and 10EK was different from another three water-soluble elements. Their concentrations were lower than that of 0K, but the concentrations of another three water-soluble elements in 10K and 10EK were higher than those of 0K. The reason why there was a decrease in the concentration in water-soluble Ca of 10K and 10EK may be relevant to the reaction that water-soluble Ca ion react with CO_2_ in water to form water-insoluble CaCO_3_. After wetting with water, there were some CaCO_3_ formed in the incubated mineral powder with and without earthworms, leading to the decrease of their water-soluble Ca. The reason why the concentration of water-soluble Ca in the mineral powder incubated by earthworms (10EK) was higher than that unincubated control (10K) was due to the weathering of mineral powder induced by earthworms. Because calcium carbonate is completely soluble in hot nitric acid solution, the concentration of water-soluble in mineral powder is only relevant to the degree of weathering and has nothing to do with the form of CaCO_3._

The observation of substrates and earthworms' gut contents under TEM indicated that all mineral grains in the intestine were wrapped into sleek pellets by intestinal mucus. The reason for this phenomenon is that the PBRP crystal is very dense and their surfaces have relatively sharp edges. In order to protect the digestive tract, earthworms produce mucus to wrap up mineral particles. These mineral particles wrapped up by mucus appeared as round pellets in the gut of earthworms.

Carpenter *et al.* suggested that the earthworms' gizzard may lead to the physical breakdown of mineral grains, and will increase the surface area open to attack and accelerate the degradation of minerals [8]. Edwards and Bohlen stated that the original coarser mineral grains are evidently comminuted by the muscular action as they pass through the earthworms' intestine [11]. However, our images of TEM showed that all PBRP in the gut of earthworms were wrapped up by mucus and formed round pellets. Thus, earthworms' gizzard played an important role in physical breakdown of PBRP, but its intestine might played a minor role in physical breakdown of PBRP as the mineral grains were wrapped up by mucus. The organic acids, digestive enzymes and microorganisms in the mucus might weather mineral grains in gut. Besides, the gut microflora and the surrounding microbes in diet substrates should have played an important role in increased degradation of PBRP.

Previous studies have shown that earthworm activity promotes organic matter decomposition significantly and enhances the nutrient release of soil organic matter [40]. Our results showed earthworms can also accelerate degradation of PBRP, indicating that they contribute nutrient release from soil minerals. Besides, they improve soil porosity by burrowing, mixing soil and enhancing aggregation [41]. Because their important role in ecosystem processes, Aristotle called them the “intestines of the earth” [42].

### Gut microbial communities of earthworms relevant to mineral degradation

The initial prepared PBRP had a very low bacterial diversity index, but other samples, including 10ES, 10GS, 0K, 10EK and 10GK, had higher values. Water is essential for microbial growth. The dry grains of originally prepared PBRP had few microorganisms due to the lack of water. The chemical weathering of minerals without the participation of water was slow. The water contents in the other samples were appropriate for the survival of microorganisms, and the nutrients from the soil or the PBRP weathered by earthworms also contributed to the microbial survival. Therefore, microorganisms were abundant in the soil after being fed to earthworms, the gut content of the earthworms fed with soil, the PBRP fed to earthworms and the gut content of the earthworms fed with PBRP.

It was noteworthy that the bacterial diversities in the guts of the earthworms fed with PBRP and the PBRP after earthworms' action were even higher than the ones in the guts of the earthworms fed with soil and the soil after earthworms' action, but the bacterial diversity of initial prepared PBRP was very low. This result indicated that there were more microorganisms surviving on the surface of PBRP in the presence of earthworms and appropriate amount of water in the mineral grains. Though PBRP had few nutrients, mineral biodegradation induced by earthworms as well as earthworms' cast and digestive juice provided nutrients for microbial growth. The existing of these microbes on the surface of mineral grains further increased the rate of mineral degradation and provide more nutrients for themselves.

The phylogenetic analysis revealed some members from *Gammaproteobacteria* were dominant in the two gut samples, but the number of shared OTUs between the five samples showed that the gut microbiota was influenced by the food source and was considerably different from that of the surrounding substrate. The results were similar to the conclusions of Knapp *et al*. who used DGGE (Denaturing gradient gel electrophoresis) to analyze the gut microflora of earthworm *Lumbricus rubellus* Hoffmeister under different feeding conditions [26]. Based on the richness of *Gammaproteobacteria* in the gut of earthworms, we supposed that some members from *Gammaproteobacteria* played an important role during the process of weathering mineral grains mediated by earthworms. The ability of *Gammaproteobacteria* in solubilizing PBR was also found by Zhao *et al*. [43]. They isolated thirty-five mineral-solubilizing bacteria from the weathered K-feldspar and soil samples and found *Gammaproteobacteria* were the dominant groups, thereby also concluding *Gammaproteobacteria* might play an important role in the process of K-feldspar weathering [43].

It usually takes 12∼20 h for the food to pass through the gut of earthworms [44]. In this feeding experiment, earthworms were fed with PBRP for ten days and thus the gut microbiota of the earthworms before using PBRP to feed had little influence on the microbial communities of the gut of earthworms and the surrounding mineral grains. The phylogenetic trees and the analysis of diversity and abundance revealed that the microbial communities in the gut of earthworms fed with PBRP and their surrounding mineral grains were very different from that of the gut of earthworms fed with soil and surrounding soil. This result suggested that the composition of the microbial communities contributing to the degradation of the PBRP was influenced by environmental conditions. The microbial diversity in the gut and the surrounding mineral grains also indicated that the degradation of mineral was caused by a combined action of many microorganisms during feeding of earthworms with PBRP. No substrate but PBRP was fed to earthworms in the feeding experiment, earthworms' living only depended on the nutriments which were released from PBRP mainly by microorganisms and these microorganisms' living depended on the nutriments from earthworms' organic matter and their weathering to PBRP. Therefore, there was a relationship of mutualistic symbiosis between earthworms and these microorganisms. An ecosystem of metabolic network for weathering PBRP was forged through their symbiosis. In this metabolic network, the mineral degradation was induced by extra-cellular polysaccharides, organic acid and other matter excreted by microbes and the digestive juice of earthworms, and the nutrients from weathered mineral were provided as a food source for earthworms and microorganisms. On the other hand, the casts and the body fluid of earthworms also provided nutrients for the microorganisms.

### Differences of bacterial communities in earthworms' gut and surrounding substrates

In UPGMA cluster analysis with unweighted UniFrac, bacteria from earthworms' gut and its surrounding substrate resemble each other, showing their similarity in bacterial diversity. Unweighted UniFrac is a qualitative measure, and it is most informative when communities differ primarily by what can live in them [39]. Because unweighted UniFrac measure considers only taxa that are present, the result of unweighted UniFrac revealed that earthworms' gut and their surrounding substrate shared similar microbiota with similar community membership, regardless of their abundance. Different surrounding substrate may be the reason that resulted in their differences in microbial community considering only the presence/absence of taxa.

Comparing to unweighted UniFrac, weighted UniFrac is quantitative measures and is ideally suited to revealing community differences that are due to changes in relative taxon abundance. In contrast, quantitative measures that account for the relative abundance of microbial lineages can reveal the effects of more transient factors such as nutrient availability [39]. The result of weighted UniFrac showed the two samples from surrounding substrate shared a similar microbial community, and the two samples from earthworms' gut shared another similar microbial community, considering the relative abundance of microbial lineages. The difference between the earthworms' gut and its surrounding substrate may be the main factor that leaded to their different microbial communities based on the relative abundance of OTUs from each clone library.

### Conclusions

Earthworms can promote the weathering of K-feldspar, which was proved by the changes in the concentrations of the water-soluble and HNO3-extractable elements released from K-feldspar grains after being digested by earthworm. They play an important role in ecosystem processes not only through their positive effects on soil structure, but also through their promotion to the nutrient cycling of ecosystem.

A higher bacterial diversity was observed in the K-feldspar grains fed to earthworms for ten days and in their gut contents, but their structure of microbial community differed from each other. The results revealed that the composition of the microbial communities related to the mineral weathering varied with environmental conditions and a large number of microorganisms played their role in the weathering process of K-feldspar weathering. Symbiosis occurred not only between earthworms and microorganisms but also among the microorganisms. An eco-system of metabolic networks for weathering K-feldspar was formed among them.
